# High-Sensitivity Cardiac Troponin as a Predictor of Adverse Events in Cardiac Sarcoidosis: A Systematic Review

**DOI:** 10.7759/cureus.97604

**Published:** 2025-11-23

**Authors:** Bader Aldhafeeri, Jafar Alayed, Ahmad Alhaj, Horiah Amer Al-Ghorbany, Abdullah Mohammed Alenezi, Ammar Ashour, Dana Alkalkili, Zaid Hasan, Dima AbuAlFool, Besan Jaber, Anas Janem, Raghad Alasali, Abdullah Altamimi

**Affiliations:** 1 Department of Pediatrics, New Jahra Hospital, Al Jahra, KWT; 2 Faculty of Medicine, Yarmouk University, Irbid, JOR; 3 School of Medicine, University of Jordan, Amman, JOR; 4 Faculty of Medicine, Helwan University, Helwan, EGY; 5 Department of Pediatrics, Farwaniya Hospital, Al Farwaniyah, KWT; 6 Faculty of Medicine, Jordan University of Science and Technology, Irbid, JOR; 7 Department of Pediatric Emergency, King Fahad Medical City (KFMC), Riyadh, SAU

**Keywords:** adverse cardiac events, cardiac bnp, cardiac sarcoidosis, cardiac troponin, high-sensitivity cardiac troponin (hs-ctn)

## Abstract

Sarcoidosis is a multisystem granulomatous disease with a higher incidence in African Americans and Scandinavians. Cardiac sarcoidosis causes arrhythmias, atrioventricular block, heart failure, and sudden death. Elevated high-sensitivity cardiac troponin T (hs-cTnT) and B-type natriuretic peptide (BNP) predict adverse outcomes despite left ventricular ejection fraction (LVEF) normalization. Corticosteroids, implantable cardioverter-defibrillators (ICDs), and angiotensin-converting enzyme (ACE) inhibitors remain key therapies, but evidence is limited, warranting a systematic review. This review searched five databases for English studies on cardiac sarcoidosis up to July 2025. Three observational studies met the patient-exposure-comparison-outcome (PECOS) criteria. Data extraction covered demographics, comorbidities, and hs-cTnT outcomes. Quality was assessed using the National Institutes of Health (NIH) tool, with narrative synthesis due to heterogeneity. This systematic review included three Japanese studies with 258 patients (142 low vs. 116 high hs-cTnT). High hs-cTnT was linked to older age, male predominance, higher BNP, lower LVEF, and worse renal function. Adverse outcomes were significantly more frequent, including cardiac death, ventricular tachycardia (VT)/ventricular fibrillation (VF), heart failure, and hospitalizations. Combined high hs-cTnT and BNP conferred the greatest risk. Imaging showed no major differences except for more right ventricular uptake in high hs-cTnT patients. This review shows that high hs-cTnT strongly predicts poor outcomes in cardiac sarcoidosis, independent of other factors. Combined with BNP, it refines risk stratification. Unlike imaging, hs-cTnT may uncover ongoing myocardial injury, supporting its role in guiding clinical management.

## Introduction and background

Sarcoidosis is a multisystem disorder characterized by the formation of non-caseating granulomas in multiple organs. The etiology of sarcoidosis remains unknown [1]. Cardiac sarcoidosis (CS) is considered the second leading cause of sarcoidosis-related death worldwide, while it is the leading cause of death among Japanese patients [2]. CS is marked by patchy, localized granulomatous inflammation of the pericardium, myocardium, and endocardium [2]. It can present with sudden cardiac death, ventricular arrhythmias, high-grade atrioventricular (AV) block, or symptoms of heart failure [2].

Sarcoidosis can affect individuals of any age or ethnicity, but its incidence is higher among African Americans and Scandinavians compared to other Caucasians [1]. Sarcoidosis often presents before the age of 50, and roughly 70% of cases present between the ages of 25 and 40, with a secondary incidence peak in women over 50 [1]. In a well-conducted five-year study in the United States, the age-adjusted annual incidence of sarcoidosis was reported as 10.9 per 100,000 among Caucasian Americans and 35.5 per 100,000 among African Americans [1]. 

Sarcoidosis is expected to affect 0.85% of Caucasian Americans, while it rises to 2.4% in African Americans over their lifetimes [1]. Elevated high-sensitivity cardiac troponin T (hs-cTnT) (>0.014 ng/mL) is an independent predictor of cardiac death and life-threatening arrhythmias in patients with CS [3]. On top of that, an increase in both hs-cTnT (>0.016 ng/mL) and B-type natriuretic peptide (BNP) (>140 pg/mL) leads to a higher risk compared to those with low levels for both biomarkers [4]. Left ventricular ejection fraction (LVEF) <40% predicts cardiac mortality, ventricular arrhythmia, and hospitalization due to heart failure [3]. LVEF improvement was noted in high and low levels of hs-cTnT, with a greater frequency of adverse effects in patients with persistently increased hs-cTnT [5]. 

This suggests that normalization of LVEF does not nullify risk in patients with chronically raised troponin. Corticosteroids are the first-line treatment for CS patients [6]. An implantable cardioverter-defibrillator (ICD) implant is indicated in patients with CS and previous sustained ventricular arrhythmias and those with LVEF ≤35%, irrespective of medical therapy and immunosuppression [6]. ICD was implanted more often in patients with high hs-cTnT levels compared to those with normal hs-cTnT levels [3]. Angiotensin-converting enzyme (ACE) inhibitors are the symptomatic heart failure therapy of choice and are used more frequently in patients with high hs-cTnT levels [3]. Current evidence on hs-cTnT in CS is limited by small single-center studies [4,5,7], inconsistent measurement protocols, and a lack of consensus on interpreting longitudinal trends [8]. No systematic review has synthesized these findings, creating a gap in clinical guidance. This review aims to evaluate the prognostic value of hs-cTnT in CS and its association with adverse cardiac events to guide clinical decision-making.

## Review

Methods

Data Collection and Search Strategy

Five databases, namely, PubMed (MEDLINE), Embase, Scopus, Web of Science, and Cochrane Library, were searched. We used a focused search strategy that included keywords and Medical Subject Headings (MeSH) terms specific to the patient population: (("high-sensitivity cardiac troponin" OR "hs-cTn" OR "cardiac troponin T" OR "cTnT") AND ("cardiac sarcoidosis" OR "heart sarcoidosis" OR "sarcoidosis myocardium")). We included all studies published up to July 25, 2025, and only included studies published in the English language.

Eligibility Criteria

After following the patient-exposure-comparison-outcome (PECOS) criteria, the inclusion criteria were as follows: (1) The population included patients with CS. (2) Since there was no intervention, the exposure was high-sensitivity cardiac troponin. (3) The comparator was patients with low or undetectable cardiac troponin. (4) Outcomes included mortality, hospitalization, ventricular tachycardia (VT), ventricular fibrillation (VF), heart failure, and device therapy. Three studies were included are observational studies (one prospective and two retrospective). We excluded case-control studies, cross-sectional studies, case reports, case series, pilot studies, commentaries, editorials, animal trials, and publications in abstract format.

Study Selection

Eligible studies that met the inclusion criteria were selected and uploaded to EndNote (Clarivate, London, United Kingdom). After removing duplicates, two authors independently screened the abstracts and full texts via Rayyan (Rayyan Systems Inc., Cambridge, Massachusetts, United States). Full-text screening was performed to further assess the relevance of the selected studies. Any disagreements were settled through discussion and reviewer consultation.

Data Extraction

Three reviewers selected the data using a standardized data extraction form. The extracted data included the country of origin, study design, and whether high- or low-sensitivity cardiac troponin assays were used. Additional variables extracted were the number of patients, mean age, gender distribution, presence of hypertension and/or diabetes mellitus, and the main reported outcomes that informed our conclusions.

Risk of Bias

We used the National Institutes of Health (NIH) Quality Assessment Tool for observational cohort and cross-sectional studies. This tool includes 14 criteria evaluating internal validity in domains such as sample representativeness, clarity of objectives, exposure and outcome measurement, and adjustment for confounders. Based on this assessment, studies were rated as good, fair, or poor.

Statistical Analysis

A meta-analysis was not performed due to marked heterogeneity in clinical and methodological characteristics among studies. Instead, we illustrated the findings narratively.

Results

Search Results

**Figure 1:**
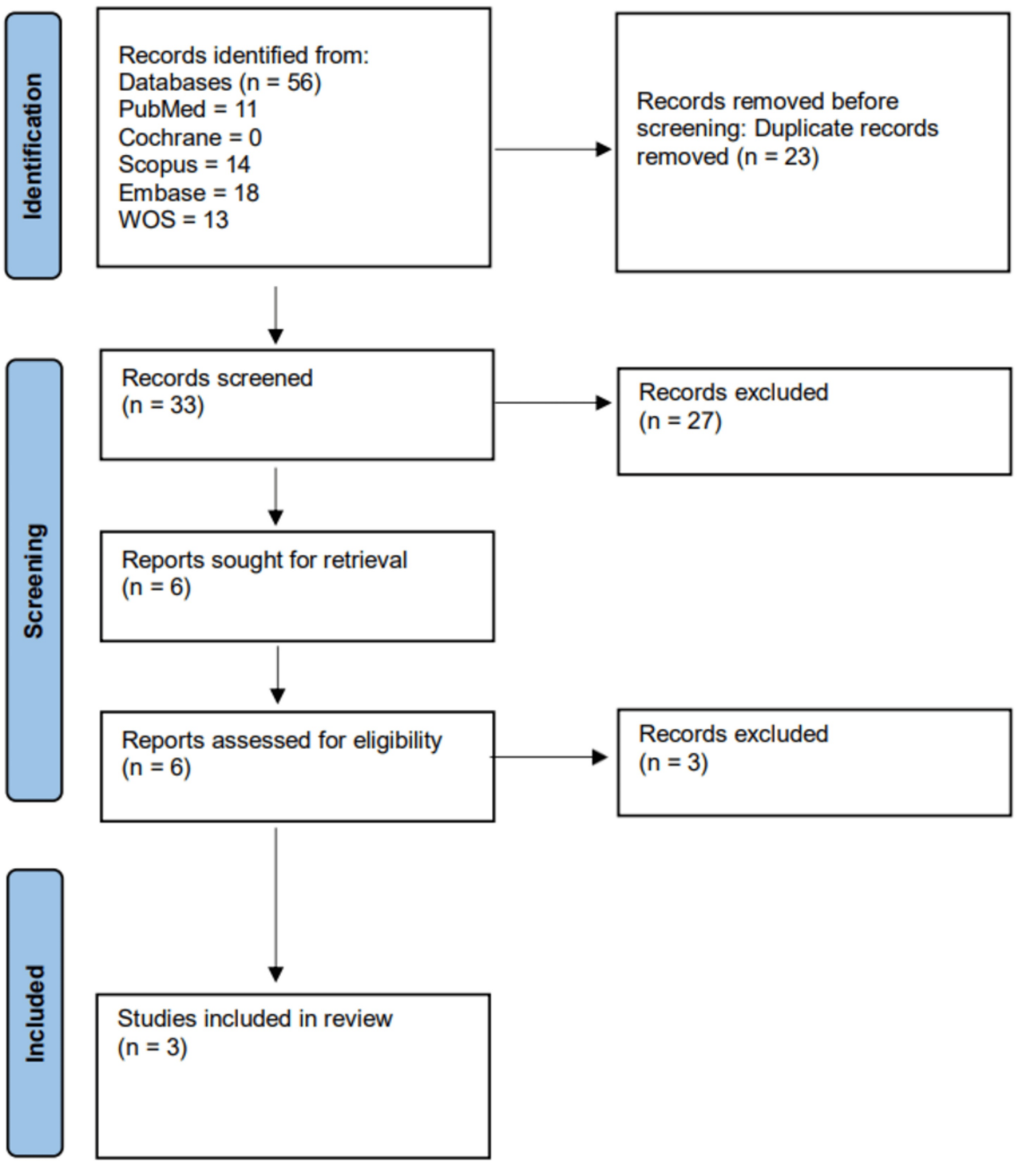
Flowchart of the selection process WOS: Web of Science

The search strategy yielded 56 studies from MEDLINE (via PubMed), Web of Science, Scopus, Embase, and the Cochrane Library. After eliminating duplicates, 33 unique records were screened by title and abstract, resulting in the exclusion of 27 studies. The remaining six full-text articles were independently reviewed, and only three studies met the inclusion criteria. A detailed overview of the search and selection process is shown in Figure 1.

Study Characteristics and Quality Assessment 

This systematic review spanned three studies from Japan [4,5,9], with a total of 258 patients averaging 62.4 years. Patients were divided into two groups: 142 patients in the low hs-cTnT group, with an average age of 59.9 years, and 116 patients in the high hs-cTnT group, with an average age of 65.3 years. The study population showed a female predominance, with 174 female patients (108 in the low vs. 66 in the high hs-cTnT group) and 84 males (34 in the low vs. 50 in the high hs-cTnT group) (Table 1).

**Table 1:** Baseline characteristics hs-cTnT: high-sensitivity cardiac troponin T; BNP: B-type natriuretic peptide; PSL: prednisolone; CS: cardiac sarcoidosis

Study ID	Country	Design	Number of patients		Mean age		Male, female		Hypertension		Diabetes mellitus		Conclusion
			Low hs-cTnT	High hs-cTnT	Low hs-cTnT	High hs-cTnT	Low hs-cTnT	High hs-cTnT	Low hs-cTnT	High hs-cTnT	Low hs-cTnT	High hs-cTnT	
Baba et al. 2025 [4]	Japan	Multicenter retrospective	52	51	59.4	65.0	9/43	23/28	18	23	14	14	High hs-cTnT associated with increased adverse events; prognostic value enhanced when combined with BNP
Kazui et al. 2023 [5]	Japan	Single-center retrospective	35	28	59	64	6/29	7/21	12	13	4	4	Higher cumulative troponin release associated with adverse outcomes despite PSL therapy
Takaya et al. 2025 [9]	Japan	Prospective observational	55	37	61	67	19/36	20/17	N/A	N/A	N/A	N/A	High hs-cTnT independently predicted cardiac death and other major events in chronic-phase CS

The quality assessment of the included studies was good, which indicates low risk of bias (Table 2).

**Table 2:** Quality assessment of the selected studies Each bar represents the proportion of responses for the 14 criteria: (N1) clear research question/objective; (N2) defined population; (N3) ≥50% participation; (N4) uniform recruitment criteria; (N5) sample size justification; (N6) exposure before outcome; (N7) adequate time frame; (N8) exposure levels analyzed; (N9) reliable exposure measures; (N10) repeated exposure assessment; (N11) reliable outcome measures; (N12) blinded outcome assessors; (N13) ≤20% loss to follow-up; and (N14) confounders measured/adjusted. *: yes; -: no; /: cannot determine

Study name	N1	N2	N3	N4	N5	N6	N7	N8	N9	N10	N11	N12	N13	N14	Total	Quality
Baba et al. [4]	*	*	*	*	-	*	/	*	*	-	*	*	*	-	10	Good
Kazui et al. [5]	*	*	*	*	-	*	*	*	*	/	*	*	*	*	12	Good
Takaya et al. [9]	*	*	*	*	*	*	*	*	*	-	*	*	*	*	13	Good

High-Sensitivity Cardiac Troponin and Its Correlation With Clinically Important Variables

Baba et al. [4] divided their study population, which consisted of 103 patients, into two populations, namely, high hs-cTnT and low hs-cTnT, according to the median value of 0.016 ng/mL. Patients with higher hs-cTnT were found to be older than those with lower levels. This was supported by both Takaya et al. [9] and Kazui et al. [5], wherein, in Kazui et al.'s findings [5], patients with a higher area under the cardiac troponin T trajectory were older with a higher BNP, while Baba et al. [4] observed the higher hs-cTnT group to have a higher prevalence of male sex, as well as lower ejection fraction. hs-cTnT levels were also found to have a positive, albeit weak, correlation with BNP levels (r=0.473; p<0.001). A negative, weak correlation with eGFR was also found (r=0.431; p<0.001), reflecting the lower renal function observed in the subgroup with high hs-cTnT. Higher BNP levels, lower LVEF, and a higher prevalence of heart failure among the high hs-cTnT group were also observed in Takaya et al.'s study population.

High-Sensitivity Cardiac Troponins and Adverse Events

In Baba et al. [4], 24 patients had primary outcomes (18 fatal ventricular arrhythmic events (FVAEs), nine all-cause deaths, and five heart failure hospitalizations). High hs-cTnT level was significantly associated with higher incidences of primary outcome and all-cause death (log-rank, p=0.017 and p=0.015, respectively), where it was also significantly associated with an increased risk of the primary outcomes (HR: 7.250 (95% CI: 2.760-19.049); p<0.001). hs-cTnT was also independently associated with a worse prognosis after adjusting for age, sex, and estimated glomerular filtration rate (eGFR) (HR: 4.368 (95% CI: 1.032-18.480)). In Kazui et al.'s cohort [5], 12 out of 63 patients experienced primary outcomes: two sudden cardiac deaths, eight VT/VF, and two events of worsening heart failure. The occurrence of primary outcomes was significantly associated with high hs-cTnT levels (p=0.017). A higher area under the cardiac troponin T trajectory was associated with a hazard ratio of 4.3 (95% CI: 1.63-11.5) for developing primary outcomes (adjustment for confounding factors, including BNP, history of VT/VF, and post-diagnosis radiofrequency ablation for VT, had no significant impact on the risk estimates). Kazui et al. [5] also measured cardiac troponin T levels a month before starting therapy with prednisolone and one month after and calculated the difference between them. None showed a significant association with the occurrence of adverse events.

In Takaya et al.'s study [9], nine cardiac deaths were observed, seven in the high hs-cTnT group and two in the low hs-cTnT group, where the high hs-cTnT subgroup was observed to have a significantly higher rate of cardiac death (log rank, p<0.01) compared to the group with the normal levels. A Cox proportional analysis also showed high hs-cTnT to be independently associated with cardiac death. Thirty-five patients, 24 from the high and 11 from the low hs-cTnT group, died or experienced cardiac events: VF occurred in one patient in the high hs-cTnT group who had been treated with an ICD, and 20 patients experienced sustained VT, 12 from the high hs-cTnT group (11 who had been treated with an ICD) and eight from the group with normal levels, all of whom had been treated with ICDs. In the high hs-cTnT group, three out of the 12 patients died suddenly, and 11 were hospitalized for heart failure, of whom one subsequently died suddenly and three subsequently died of heart failure. Of the eight patients with normal hs-cTnT levels, one died suddenly, and three were hospitalized for heart failure, of whom one subsequently died of heart failure. Patients with high hs-cTnT showed significantly higher rates of cardiac death, VF, sustained VT, or hospitalization for heart failure compared with the normal hs-cTnT group (log rank, p<0.01). Five or more ventricular tachyarrhythmias occurred in eight patients with high hs-cTnT and four patients with normal levels, where the rate of tachyarrhythmias was significantly higher in the high hs-cTnT group (p=0.04). Two or more hospitalizations for heart failure occurred in seven patients with high and one with normal hs-cTnT, where the rate of hospitalization was significantly higher in the hs-cTnT group (p<0.01). The rate of fatal cardiac events (cardiac death, VF, sustained VT, including ICD) was significantly higher in patients with high hs-cTnT than those with normal levels (log-rank, p<0.01). The mean hs-cTnT level was 0.020±0.021 ng/mL at the initial measurement and 0.022±0.020 ng/mL at the latest measurement, where there was no significant difference between them. Similarly, among the high hs-cTnT group, with a total of 37 patients, no significant difference was found between the initial and final measurements (0.035±0.026 ng/mL to 0.038±0.024 ng/mL; Pp=0.66), while in the group with normal hs-cTnT levels which consisted of 55 patients, the levels were higher in the latest measurement compared to the initial one, but the increase was not remarkable (0.009±0.004 to 0.012±0.006 ng/mL; p<0.01).

High-Sensitivity Cardiac Troponins and LVEF

Kazui et al. [5] observed a relationship between longitudinal cardiac troponin T levels and changes in LVEF, where LVEF changed from 44 (33-51%) to 42 (38-52%; p=0.003) in the lower cardiac troponin T trajectory per month group, while the improvement was 42 (33-52%) to 48 (43-54%; p=0.053) for the higher group. No significant difference was found between the groups. Lower LVEF was observed in Takaya et al.'s study among the high hs-cTnT group.

High-Sensitivity Cardiac Troponins, BNP Levels, and Risks for Cardiac Events

When Baba et al. [4] divided the study population into three groups according to combined median levels of hs-cTnT and BNP, the subgroup with both high hs-cTnT and high BNP values was significantly associated with a higher incidence of the primary outcome compared to the other two groups (high hs-cTnT and low BNP and low hs-cTnT and low BNP). In addition, patients in this subgroup also showed a 3.49-fold increased risk of primary outcomes compared with patients with both low hs-cTnT and BNP levels. This finding is consistent with Takaya et al. [9], who reported that patients with both elevated hs-cTnT and elevated BNP had the highest rates of cardiac death, VF, sustained VT, or hospitalization. In contrast, these adverse outcomes were lowest among patients with normal hs-cTnT and low BNP levels (log-rank, p<0.01).

High-Sensitivity Cardiac Troponins and Imaging

Baba et al. [4] found no significant difference between the groups in gallium-67 (Ga)-scintigraphy, fluorodeoxyglucose positron emission tomography (FDG-PET), or cardiac magnetic resonance imaging (CMRI). Kazui et al. [5] likewise found no significant difference in LVEF and imaging findings. Takaya et al. [9] reported that Ga-scintigraphy, FDG-PET, and CMRI showed no significant differences between the patient groups. However, Takaya et al. [9] did find more frequent right ventricular uptake in the high hs-cTnT group compared to the lower one (24% vs. 9%; p=0.04). No correlation between hs-cTnT and ACE was found.

Discussion

This is the first systematic review to be conducted on the evidence of hs-cTnT as a predictor of adverse events in CS. In three Japanese cohort studies with a total of 258 patients, higher hs-cTnT levels appear to be more prone to cardiac adverse events, such as heart failure hospitalization, cardiac death, and ventricular tachyarrhythmias [4,5,9]. The results of these studies demonstrated the potential of hs-cTnT as a non-invasive tool to help detect patients at higher risk of developing cardiac adverse events. In these studies, hs-cTnT levels were generally higher in patients who were older and male and in those with indicators of myocardial stress such as elevated BNP levels and reduced LVEF [4,5,9]. After adjusting for conventional risk factors, including sex, age, and eGFR, elevated hs-cTnT levels were independently associated with adverse events with hazard ratios ranging from 4.368 to 7.250 [4,5]. The ability of hs-cTnT to predict adverse cardiac events doesn't get affected by the way hs-cTnT levels were defined, whether by an absolute threshold, a median split, or a trajectory overtime. Patients with low hs-cTnT and low BNP had the best outcomes, while those with high levels of both markers experienced the worst outcomes; this makes the relationship between hs-cTnT and BNP notable [4]. Interestingly, hs-cTnT levels do not appear to be related to differences in imaging findings, such as FDG-PET uptake, Ga-scintigraphy, or CMRI, except for more frequent right ventricular uptake reported in one study [5]. This indicates that hs-cTnT may help detect myocardial injury that is not visible on imaging.

Comparison With Other Studies

Our findings are supported by evidence from larger international cohorts. The Mayo Clinic study by Kolluri et al. confirmed that troponin T is an independent predictor of adverse outcomes, including death, left ventricular assist device (LVAD) implantation, and heart transplantation, even after adjustment for LVEF [8]. Similarly, Kandolin et al. observed that troponin levels decreased rapidly after corticosteroid initiation, reinforcing their usefulness as indicators of treatment response [10]. Taken together, these external findings are consistent with our results and further support the role of hs-cTnT as both a prognostic marker and a tool for monitoring disease activity in CS. Our findings are consistent with previous studies on biomarkers in CS, where BNP and N-terminal pro-B-type natriuretic peptide (NT-proBNP) have been identified as predictors of poor outcomes, as they reflect increased cardiac strain [11]. hs-cTnT adds another layer of information by showing ongoing heart muscle injury [12]. When both markers are elevated, as reported by Baba et al., Takaya et al., and Kolluri et al., patients are at the highest risk of adverse events [4,8,9]. Compared with advanced imaging techniques, troponin represents a practical, affordable, and repeatable biomarker [8]. Although CMRI and FDG-PET remain the gold standards for diagnosing and monitoring, their limited availability and high cost restrict their routine use [5,8,9]. In contrast, troponin testing is widely accessible in daily practice and can be used for ongoing follow-up [8]. Importantly, it may complement imaging by detecting active myocardial injury even when new structural changes are not apparent [4,5,7,9]. Finally, our findings fit with what has been reported in other inflammatory heart diseases [7]. Recent studies show that troponin is a reliable marker of ongoing heart muscle inflammation, with prognostic value that goes beyond sarcoidosis [8,10]. Using hs-cTnT in the care of CS patients is therefore in line with broader practices in myocarditis management [7].

Interpretation and Possible Explanations

The three studies included in the systematic review consistently show that hs-cTnT levels are a powerful and independent predictor of adverse events in patients with CS [4,5,9]. The research further identified that these correlations remained even after adjustment for traditional risk factors, including age, sex, renal function, and BNP level. The prognostic utility of troponin is also supported by larger cohort studies such as Kolluri et al. [8], in which troponin T was a predictor of a composite outcome of death, LVAD implantation, or heart transplantation (HR 1.06 per 0.01 ng/mL; p=0.006) even after adjustment for ejection fraction [8]. Based on current scientific knowledge, there may be multifactorial explanations for this correlation. The typical symptoms of CS are inflammation of the myocardium and granuloma formation, being able to cause direct damage to the cardiomyocytes [13]. The biomarker hs-cTnT is extremely sensitive and specific to myocardial damage and is released into circulation when the damage occurs [14]. Baba et al. observed a weak positive correlation between hs-cTnT and BNP (r=0.473; p<0.001) and a weak negative correlation with eGFR (r=0.431; p<0.001), suggesting that the biomarker reflects a combination of myocardial stress and injury, possibly worsened by impaired renal clearance in some patients [4]. According to the two studies of Baba et al. (HR: 3.49; 95% CI: 1.23-9.88) and Takaya et al., patients with both high hs-cTnT and high BNP had the most adverse events, confirming the notion that both myocardial injury and hemodynamic stress indicate a poorer prognosis [4,9]. This is reinforced by Kolluri et al.'s work, where NT-proBNP was significantly higher in definite CS compared to probable CS (3200 pg/mL vs. 1100 pg/mL; p=0.02) and both NT-proBNP and BNP correlated with the secondary end point of cardiac hospitalization-free survival [8]. Kazui et al.'s research findings indicate that a lone cardiac troponin T measurement before or one month after prednisolone administration was not a strong predictor of outcomes. The longitudinal trend of cardiac troponin T release as area under the curve over time, however, was very strongly associated with the main outcome (HR: 4.3; 95% CI: 1.63-11.5), suggesting that the chronic burden of myocardial injury is a greater cause of bad outcomes than the acute burden at presentation [5]. This ongoing chronic myocardial injury often leads to progressive replacement fibrosis, ventricular dysfunction, and the creation of an arrhythmogenic substrate presenting as heart failure hospitalization, ventricular arrhythmias, or death. Besides, the quest for imaging of CS without the help of the biomarker has been going on for a long time. Despite its strong prognostic value, hs-cTnT showed no consistent correlation with imaging markers of CS. Baba et al., Kazui et al., and Takaya et al. all reported that there were no significant differences between high and low hs-cTnT groups in findings from Ga-scintigraphy, FDG-PET, or late gadolinium enhancement on CMRI [4,5,9]. Takaya et al. did find more frequent right ventricular uptake on initial imaging in the high hs-cTnT group (24% vs. 9%; p=0.04), which may reflect a greater inflammatory injury burden [9]. The poor correlation that exists overall between imaging and hs-cTnT may stem from the fact that the imaging techniques assess different components of the disease, that is, inflammation (FDG-PET), fibrosis (late gadolinium enhancement on** **CMRI), or stable activity (Ga-scintigraphy), whereas hs-cTnT is a direct, quantitative measure of ongoing cardiomyocyte necrosis. This difference also indicates hs-cTnT's potential as a complementary biomarker, as it offers prognostic information that is not identifiable by imaging alone.

Strengths of This Review

This systematic review is supported by various key strengths. First, it synthesizes evidence from all three studies, which focus on the prognostic role of hs-cTnT in CS, thereby summing up to 258 patients. This accounts for a significant proportion of published data on this highly specific biomarker in a rare disease field. Second, the review makes use of results from the studies that investigated hs-cTnT in various clinical conditions, providing a more comprehensive review. Baba et al. [4] focused on the biomarker's application at the time of diagnosis, Kazui et al. [5] revealed the biomarker's longitudinal trajectory after treatment onset, and Takaya et al. [9] were concerned with its use in the chronic phase after medical therapy was established. This evidence triangulation across the spectrum of CS strengthens the conclusion that hs-cTnT is a robust prognostic biomarker regardless of the clinical phase. Although differing in study design (prospective vs. retrospective) and in particular endpoints and sample size, the three studies together consistently identified statistically significant associations between elevated levels of hs-cTnT and increased risk for major adverse cardiac events, including all-cause death, cardiac death, ventricular arrhythmias, and heart failure hospitalization. Thus, this consistency markedly contributes to the validity and further generalizability of the conclusions of the review.

Clinical Implication

Importantly, the clinical implications of these findings are substantial. The reproducible link between elevated hs-cTnT and adverse outcomes suggests that this simple, widely available blood test can enhance clinical risk assessment and guide management strategies in CS. Because predicting arrhythmic or heart failure events in these patients remains challenging, incorporating hs-cTnT into risk models may allow the earlier identification of high-risk individuals who could benefit from intensified monitoring, prophylactic device implantation, or escalation of immunosuppressive therapy [15]. Furthermore, serial hs-cTnT monitoring could provide a dynamic assessment of treatment response, aiding in the titration of corticosteroids or other immunomodulatory agents. Compared with advanced imaging modalities, troponin testing is inexpensive, reproducible, and feasible for longitudinal follow-up, making it an attractive adjunct in routine care [8].

Limitations

Although the findings across studies were consistent, there are some important limitations that should be considered. First, all included studies were conducted in Japan, which limits the generalizability to other populations with different demographic and genetic backgrounds. Second, the total sample size was relatively small, which may limit the precision of risk estimates. Third, the studies differed in their design, the cut-off values used for hs-cTnT, and the outcomes they measured, making direct comparisons difficult. Lastly, there is still little evidence on how changes in hs-cTnT levels over time relate to prognosis, so the value of serial measurements remains unclear.

## Conclusions

This systematic review shows that a high level of hs-cTnT is strongly associated with the worst outcomes in CS, regardless of other clinical factors. When combined with BNP, it helps further distinguish patients at higher risk. Interestingly, hs‑cTnT may reveal ongoing myocardial injury that imaging does not detect. Using hs-cTnT in everyday clinical practice could improve how we assess risk and make treatment plans for patients with CS.
